# A comprehensive study of small non-frameshift insertions/deletions in proteins and prediction of their phenotypic effects by a machine learning method (KD4i)

**DOI:** 10.1186/1471-2105-15-111

**Published:** 2014-04-17

**Authors:** Carlos Bermejo-Das-Neves, Hoan-Ngoc Nguyen, Olivier Poch, Julie D Thompson

**Affiliations:** 1 ICube Laboratory and Strasbourg Federation of Translational Medicine (FMTS), University of Strasbourg and CNRS, Strasbourg, France; 2 Département de Biologie Structurale et Génomique, IGBMC (Institut de Génétique et de Biologie Moléculaire et Cellulaire), CNRS/INSERM/University of Strasbourg, Illkirch, France

**Keywords:** Genotype-phenotype relationship, Insertion/deletion, Machine learning, Indeuctive logic programming

## Abstract

**Background:**

Small insertion and deletion polymorphisms (Indels) are the second most common mutations in the human genome, after Single Nucleotide Polymorphisms (SNPs). Recent studies have shown that they have significant influence on genetic variation by altering human traits and can cause multiple human diseases. In particular, many Indels that occur in protein coding regions are known to impact the structure or function of the protein. A major challenge is to predict the effects of these Indels and to distinguish between deleterious and neutral variants. When an Indel occurs within a coding region, it can be either frameshifting (FS) or non-frameshifting (NFS). FS-Indels either modify the complete C-terminal region of the protein or result in premature termination of translation. NFS-Indels insert/delete multiples of three nucleotides leading to the insertion/deletion of one or more amino acids.

**Results:**

In order to study the relationships between NFS-Indels and Mendelian diseases, we characterized NFS-Indels according to numerous structural, functional and evolutionary parameters. We then used these parameters to identify specific characteristics of disease-causing and neutral NFS-Indels. Finally, we developed a new machine learning approach, KD4i, that can be used to predict the phenotypic effects of NFS-Indels.

**Conclusions:**

We demonstrate in a large-scale evaluation that the accuracy of KD4i is comparable to existing state-of-the-art methods. However, a major advantage of our approach is that we also provide the reasons for the predictions, in the form of a set of rules. The rules are interpretable by non-expert humans and they thus represent new knowledge about the relationships between the genotype and phenotypes of NFS-Indels and the causative molecular perturbations that result in the disease.

## Background

A major goal in human genetics is to understand the links between the presence of genetic variations, including Single Nucleotide Polymorphisms (SNPs), insertions/deletions (Indels), Copy Number Variants (CNV), recombination events, etc. and individual or population characteristics, risk of disease or response to the environment. This requires the characterization and analysis of the type and distribution of the variations in human populations and/or each individual to understand how a specific genetic landscape can influence human health and behavior [1-6].

Recently, with the development of Next Generation Sequencing (NGS) techniques [7], the available information related to genetic human variation has evolved rapidly, resulting in overwhelming volumes of data. For example, the dbSNP database (build 138) [8] contains about 62 million SNPs and 11 million Indels. Half a million of these observed SNPs are found within an exon and modify a single amino acid. These are known as non-synonymous SNPs (nsSNPs) and are the most frequent cause of Mendelian diseases since they alter the protein’s function [9].

Insertions and/or deletions, commonly known as Indels, are the second most frequent type of human variation [10]. When an Indel occurs within an exon, it can be either frameshifting (FS) or non-frameshifting (NFS): FS-Indels either modify the complete C-terminal region of the protein or result in premature termination of translation, while NFS-Indels involve multiples of three nucleotides, leading to the insertion/deletion of one or more amino acids [11]. The importance of Indels in the human genome has emerged recently from various studies [11-13]. More specifically, small Indels (less than 500 bp or more usually less than 200 bp) are the second most frequent cause of Mendelian diseases [9] and represent some of the least well-characterized and least understood variants in the human genome. This is due in part to the fact that they are difficult to identify accurately during NGS calling [14].

To predict the functional impact of sequence variation, numerous tools have been developed recently, mainly focusing on nsSNPs and Mendelian diseases [15]. These tools share some common features, notably: i) each variant is classified according to a two state category: neutral (often designated as Variant of Uncertain Significance) or disease-causing (deleterious) and annotated with a certain number of parameters, ii) a model is created using a machine learning technique (Support Vector Machine, Bayesian network, Inductive Logic Programing, etc.) and criteria, scores or rules are defined to distinguish known neutral variants from known deleterious ones, iii) the criteria, scores or rules are then used to predict the status of unknown variants. Among the numerous methods available, SIFT [16] and Polyphen [17] are the most widely used, since they provide good accuracy (around 80%) for the prediction of nsSNPs involved in diseases. The PROVEAN [18] method uses a scoring metric to predict the pathogeneticity of SNPs and NFS-Indels based on sequence similarity before and after the introduction of an amino acid variation. Two tools have also been published that are designed specifically to predict the effect of Indels: SIFT-indel [19,20], and DDIG-in [21].

During the last few years, we have developed an integrated framework, SM2PH [4], dedicated to the study and prediction of human genetic variation. SM2PH is built around a knowledge base, which provides unified access to diverse information associated with any human protein (pathway, tissue expression, interactions, evolution, etc.) and facilitates the integrated study of the structural and functional impacts of nsSNPs and their phenotypic effects. The framework also incorporates the MSV3d [22] database of known missense variants in all human proteins for which a 3D structure template is available. The human missense variants in MSV3d are mainly retrieved from the dbSNP [8] and SwissVar [23] databases and are classified into 2 categories: disease-causing variants associated with OMIM [24] diseases and Variants of Uncertain Significance (VUS). Each missense variant is then characterized using a large set of structural, functional and evolutionary parameters. Finally, the information and data model in MSV3d is exploited by the KD4v [25] system to predict the pathogenicity of nsSNPs based on a set of rules generated by Inductive Logic Programming (ILP) [26].

Here, we describe the extension of the SM2PH framework to include NFS-Indels. This study is composed of two parts: (i) the collection, annotation and comparative study of a reference set of disease causing and neutral NFS-Indels and (ii) the design of a machine learning method (KD4i), which is able to predict the effects of NFS-Indels and shed light on the molecular mechanisms underlying their pathogenicity.

First, the knowledge base in the SM2PH framework was extended to include a large data set of 2163 NFS-Indels, including 757 disease-causing variants and 1406 neutral or unknown variants. These variants were then annotated with an extensive set of parameters, specifically designed to describe the diverse structural, functional and evolutionary characteristics of NFS-Indels. We then performed a statistical analysis to study the general characteristics of disease-causing and neutral NFS-Indels.

Second, an Inductive Logic Programming (ILP) machine learning approach was developed to predict disease-causing NFS-Indels. ILP systems allow learning with much richer representations than many machine learning methods and the complex relationships learned are described as logic programs. In KD4i, the logic programs are provided as a set of rules that are easy to comprehend by the user. Thus, KD4i is able, not only to make a prediction, but also to give the reasons for the prediction, i.e. to generate new knowledge about the causative molecular perturbations, such as the disruption of catalytic residues, binding sites or post-translational modifications, that result in disease.

## Methods

### Data set collection

The reference set of NFS-Indels was constructed using the same approach as that described in the article presenting the PROVEAN method [18]. First, NFS-Indels were collected from the UniProtKB/Swiss-Prot database [27], and annotated in-house as deleterious, neutral or unknown, based on keywords found in the UniProtKB annotations (e.g. variants described using words such as “inhibit'', ''affect'', etc. were classified as deleterious). Second, this set was enriched with NFS-Indels retrieved from the 1000 Genomes Project database [28]. Variants with average allele frequencies of >10% were collected and could thus be considered as common, i.e. non pathogenic or neutral, in the human population.

All the variants were then mapped to the human protein sequences in the SM2PH knowledge base. This resulted in a data set containing a total of 2163 NFS-Indels, mapped to 1535 distinct proteins. Of these, 1406 NFS-Indels were defined as polymorphisms/neutral (147 insertions and 1259 deletions) and 757 as disease-causing/deleterious (108 insertions and 649 deletions). Then, 757 neutral NFS-Indels (101 insertions and 657 deletions), were randomly selected from the pool of neutral NFS-Indels. Thus, the final data set was balanced for positive/negative examples (i.e. neutral/disease-causing NFS-Indels), an important characteristic to ensure appropriate statistical analysis and quality learning. The variants used in the final data set are provided in Additional file 1.

### Dataset annotation

The annotations used to characterize the NFS-Indel data set were either extracted from the SM2PH knowledge base or calculated using existing tools for protein structural analysis and in-house developed Python scripts. The SM2PH knowledge base contains high quality multiple sequence alignments for all human proteins, which are annotated with structural and functional parameters derived from MACSIMS (Multiple Alignment of Complete Sequences Information Management System). MACSIMS combines knowledge-based methods with complementary *ab initio* sequence-based predictions to extract valuable information from multiple alignments [29]. These parameters are described in more detail in [25] and on the SM2PH help pages (decrypthon.igbmc.fr/sm2ph/cgi-bin/help). This information was complemented by additional structural parameters, including secondary structures and residue solvent accessible surface area (RSA) calculated using Spine-X [30].

The different structure, functional and evolutionary parameters are shown in Table 1 and described in detail below.

**Table 1:** Parameters used for Indel annotation with their defined values and the source of the data

**Class**	**Parameters**	**In final method?**	**Values**	**Source**
Conservation	Conserved residue	Yes	Yes/No	MACSIMS via SM2PH
	Block	Yes		
Functional	Pfam domain	Yes	Yes/No	MACSIMS via SM2PH
	Prosite motif	No		
	Uniprot domain	Yes		
Physico-chemical properties (average)	Volume	No	See table 2	In-house
	Hydrophobicity	No		
	Polarity	Yes		
	Charge	No		
Physico-chemical properties (total)	Volume	Yes	See table 2	In-house
	Hydrophobicity	Yes		
	Polarity	No		
	Charge	No		
Local perturbation in site (average)	Volume	Yes	−2 to +2*	In-house
	Hydrophobicity	Yes		
	Polarity	No		
	Charge	No		
Local perturbation in environment (average)	Volume	No	−2 to +2*	In-house
	Hydrophobicity	No		
	Polarity	No		
	Charge	No		
Local perturbation in region (average)	Volume	No	−2 to +2*	In-house
	Hydrophobicity	No		
	Polarity	No		
	Charge	No		
Local perturbation in site (total)	Volume	Yes	−2 to +2*	In-house
	Hydrophobicity	Yes		
	Polarity	No		
	Charge	No		
Local perturbation in environment (total)	Volume	No	−2 to +2*	In-house
	Hydrophobicity	No		
	Polarity	No		
	Charge	No		
Local perturbation in region (total)	Volume	No	−2 to +2*	In-house
	Hydrophobicity	No		
	Polarity	Yes		
	Charge	No		
Structural	Disorder	Yes	Structured (probability of disorder P < 0.4)	Spine-D
			Semi-disorder (0.4 < P < 0.7)	
			Disorder (P > 0.7)	
	RSA Secondary structure	Yes	Fully buried (RSA value (R_v_ < 30)	Spine-D
			Buried (30 < R_v_ < 60)	
			Intermediate (60 < R_v_ < 90)	
			Exposed (90 < R_v_ < 120)	
			Fully exposed (R_v_ > 120)	
	Secondary structure Relative Indel Position	Yes	Coil	Spine-D
			Helix	
			Strand	
			Two (if NFS-Indel is in the transition zone between a strand/helix and coil)	
Others	Relative Indel Position	Yes	N-terminal	In-house
			Middle	
			C-terminal	
	Indel length	Yes	One	In-house
			More than one	
	Presence of Proline	No	Yes/No	In-house
	Presence of Glycine	No	Yes/No	In-house

The column ‘In final method?’ indicates whether the parameter is used in the final ILP rule set for prediction of deleterious NFS-Indels. *The numerical values range from −5 to +5 but, in order to reduce computational cost, we have regrouped values higher than ±2 into the semantic category two or more/two or less.

#### Conservation

It is generally thought that conserved sites in proteins have important functional or structural roles [31]. The conservation categories for a given NFS-Indel position are extracted from MACSIMS via the SM2PH knowledge base. We used two different annotations: conserved residues and conserved ‘core blocks’, i.e. sequence segments that are conserved in subfamilies of the multiple alignments.

#### Functional annotations

It is assumed that changes at important functional sites in a protein will have major effects on its function. We therefore identify the presence of NFS-Indels in known functional sites, including domains extracted from the Pfam protein family database [32], motifs from the Prosite database [33], domains from the UniProt database, as well as the regions annotated by MACSIMS.

#### Physico-chemical properties

These parameters have been shown previously to be important for nsSNP classification [25]. Among the numerous physico-chemical properties associated with an amino acid, we have chosen four important ones: volume, charge, hydrophobicity and polarity. For a given NFS-Indel, we calculate parameters as follows:

For each amino acid in the NFS-Indel, the values for each property are translated into numerical categories (Tables 2, 3). Two parameters are then associated with each NFS-Indel: the average and the total of the individual amino acid properties. Finally, since we use a semantic algorithm for learning (see next section), the average and total values are classified into different semantic categories (Table 2).

**Table 2:** Semantic categories of the 5 physico-chemical properties: volume, hydrophobicity, charge, and polarity

**Physico-chemical property**	**Semantic categories**	**Numeric categories**	**Real values**
Volume [34]	Very small	0	60-90 Å^3^
	Small	1	108-117 Å^3^
	Medium	2	138-154 Å^3^
	Large	3	162-174 Å^3^
	Very large	4	189-228 Å^3^
Hydrophobicity [35]	Hydrophilic	0	−55 to −14
	Neutral	1	−10 to 13
	Hydrophobic	2	41 to 63
	Very hydrophobic	3	74 to 100
Polarity [36]	Polar	0	
	Apolar	1	
Charge [36]	Negative	−1	
	Neutral	0	
	Positive	1	

**Table 3:** Annotation of amino acids based on classified values of 4 physico-chemical properties: volume, hydrophobicity, charge, and polarity

**Amino Acid**	**Volume**	**Hydrophobicity**	**Charge**	**Polarity**
A	1	2	0	1
C	2	2	0	1
D	2	0	−1	0
E	3	0	−1	0
F	5	3	0	1
G	1	1	0	1
H	3	1	1	0
I	4	3	0	1
K	4	0	1	0
L	4	3	0	1
M	4	3	0	1
N	2	0	0	0
P	2	0	0	1
Q	3	1	0	0
R	4	0	1	0
S	1	1	0	0
T	2	1	0	0
V	3	3	0	1
W	5	3	0	0
Y	5	2	0	0

#### Local perturbation

In order to characterize the local perturbation induced by the NFS-Indel, we have introduced a set of original parameters, expressed as the difference between the physico-chemical properties of the NFS-Indel residues (described in Table 3) with respect to (i) the site (the amino acids that take the place of a deletion, or the original amino acids at the position of an insertion), (ii) the environment, consisting of the n (equal to the length of the NFS-Indel) flanking amino acids of a NFS-Indel or (iii) the region (twice the length of the environment). The local perturbation parameters are then defined as:

$$\mathrm{Perturbation}\left(\mathrm{average}\right)={\bar{x}}_{s}-{\bar{x}}_{i}$$

Wherex=volume,hydrophobicity,polarityorcharge

$$\bar{x_{i}}=\mathrm{average}\;\mathrm{property}\;\mathrm{of}\;\mathrm{the}\;\mathrm{NFS}-\mathrm{Indel}$$

$${\bar{x}}_{s}=\mathrm{average}\;\mathrm{property}\;\mathrm{of}\;\mathrm{the}\;\mathrm{local}\;\mathrm{sequence}\;\left(\mathrm{site},\mathrm{environment}\;\mathrm{or}\;\mathrm{region}\right)$$

And:

$$\mathrm{Perturbation}\left(\mathrm{total}\right)=\sum x_{s}-\sum x_{i}$$

Wherex=volume,hydrophobicity,polarityorcharge

$$\sum x_{i}=\mathrm{total}\;\mathrm{of}\;\mathrm{the}\;\mathrm{properties}\;\mathrm{for}\;\mathrm{the}\;\mathrm{amino}\;\mathrm{acids}\;\mathrm{in}\;\mathrm{the}\;\mathrm{NFS}-\mathrm{Indel}$$

$$\sum x_{s}=\mathrm{total}\;\mathrm{of}\;\mathrm{the}\;\mathrm{properties}\;\mathrm{for}\;\mathrm{the}\;\mathrm{local}\;\mathrm{sequence}\;\left(\mathrm{site},\mathrm{environment}\;\mathrm{or}\;\mathrm{region}\right)$$

#### Functional annotations

• *Disorder Probability:* Disordered regions in proteins are structurally flexible and hence more permissive to modification by micro-insertion or micro-deletion. Nevertheless, despite their lack of a well-defined globular structure, the disordered regions are known to be involved in many basic functions in molecular recognition and macromolecular assemblies and are frequently associated with signal transduction, cell-cycle regulation and transcription, for example [37]. Here, we define a disordered region as having a Spine-D disorder probability >0.7.

• *Relative solvent accessible surface area (RSA):* RSA is defined as the solvent accessible surface area of a residue in a protein normalized by the accessible surface area of the residue in its “unfolded” state [38]. It has been suggested that the environments around protein residues may affect their functions or their propensities for different structures [39] and therefore, amino acids may behave differently when they are buried or on the surface of the protein.

• *Secondary structure location*: The NFS-Indel location is defined as in an alpha helix, a beta sheet or a loop. Our hypothesis is that NFS-Indels may be more deleterious if they occur within secondary structures, especially in the case of beta sheets, since the loss of a single strand is likely to disrupt the overall structure.

#### Relative indel position (RIP)

It has been shown in a study devoted to the analysis of a genome from a healthy individual [40] that NFS-Indels occur more frequently in the N/C-terminal regions of proteins. The authors hypothesized that this may be due to higher selective pressure in the central part of the protein. Therefore, NFS-Indels occurring in this region may be deleterious. We defined the RIP as the ratio between the position of the NFS-Indel and the length of the protein. The N-terminal region is then defined as the first 10% of the protein and C-terminal region as the last 10%.

#### Indel length

We used the definition of a ‘small’ NFS-Indel given in [40], as being in the range of 3–24 base pairs (i.e. 1–8 amino acids). In fact, 62% of the NFS-Indels were < =6 base pairs and longer NFS-Indels become increasing rare, and NFS-Indel of 24 bp (8 amino acids), represent only 3%. We limited our study to NFS-Indels ranging from 3–18 base pairs for which enough data is available. We hypothesize that longer NFS-Indels are more disruptive than shorter ones for the protein structure.

#### Presence of proline/glycine

It has been shown that Proline and Glycine play a role in protein folding. For example, Glycine presents faster rate constants for contact formation than any other amino acid, as expected from its increased backbone flexibility due to the lack of a C_β_-atom. The presence of a Proline residue leads to more complex dynamics in the process of contact formation, and this amino acid is also a strong α-helix breaker [41].

### Machine learning strategy

Machine learning, a branch of artificial intelligence, concerns the construction and study of computational systems that can learn from data. The development of a machine learning tool requires three steps. The first one is to obtain a data set that is large, representative and error-free, in order to ensure accurate automated learning. The second is to develop a strategy for the characterization of the objects or examples contained in this data set. This is called annotation of the data set and includes the parameters used to characterize the examples in the data set, as well as the categories or classes that are to be learned (in this case, deleterious or neutral variants). These two steps are done in the two previous paragraphs ‘Data set collection’ and ‘Data set annotation’. The third is to design an efficient machine learning strategy. This includes the pre-selection of the parameters that will be used during learning and the choice and optimization of the learning algorithm [42].

Here, we have used a machine learning algorithm called Inductive Logic Programming (ILP), which infers hypotheses from experience (inductive learning) by means of logic programming. The approach is based on positive and negative examples, which combined with background knowledge, can be used to infer a hypothesis. Positive and negative examples are objects (here, NFS-Indels) from a training set that satisfy a condition (here, are deleterious or neutral) and the background knowledge consists of the set of parameters for these objects. ILP searches for a combination of parameter values that covers the maximum number of positive examples and the minimum number of negative examples. Such combinations are called ‘rules’. The process is then repeated until all positive examples are covered by at least one rule.

We used the ILP algorithm implemented in Aleph [26]. Aleph allows the user to set a number of program options, including the minimum number of positive examples (minpos) and the maximum number of negative examples for each rule (noise). Then ILP evaluates each rule based on the difference between the number positive examples covered and the number of negative examples. If the minpos and noise constraints are satisfied the rule is added to the hypothesis space. In the experiments described in the Results section we set minpos = 6 and noise = 0, in order to eliminate false positive predictions.

The rules produced by the ILP algorithm can be used to predict the status of unknown objects, i.e. to predict whether an unknown NFS-Indel is deleterious or neutral. To evaluate the prediction power of our algorithm, we used a 10-fold cross-validation strategy. Specifically, we randomly split the data set into 10 parts: 9 parts were used to train the ILP algorithm and 1 part was used to estimate the prediction performance of the method. The performance was assessed using the statistical parameters shown in Table 4 and defined in [42]. This process was repeated 10 times.

**Table 4:** Statistical parameters used to assess the prediction performance of the ILP method

		**Real**		
		**Positive**	**Negative**	
Prediction	Positive	Tp	Fp	Precision TpTp+Fp
	Negative	Fn	Tn	NPV TnTn+Fn
		Sensitivity TpTp+Fn	Specificity TnTn+Fp	MCC $\frac{\mathrm{Tp}*\mathrm{Tn}-\mathrm{Fp}*\mathrm{Fn}}{\sqrt{\left(\mathrm{Tp}+\mathrm{Fp}\right)\left(\mathrm{Tp}+\mathrm{Fn}\right)\left(\mathrm{Tn}+\mathrm{Fp}\right)\left(\mathrm{Tp}+\mathrm{Fn}\right)}}$
		Accuracy: Tp+TnTp+Tn+Fp+Fn		

T=True, F=False, p=Positive, n=Negative, NPV=Negative predictive value, MCC=Matthews correlation coefficient.

### Parameter selection

Before running Aleph, we performed a pre-selection of parameters to avoid the effects of the ‘curse of dimensionality’ [43] due to the large number of parameters and the relatively small number of examples in the data set. Several approaches have been proposed for dimensionality reduction, including tests that are independent of the machine learning algorithm, such as Principal Component Analysis (PCA) [44], which we used initially to investigate the level of redundancy in our parameter set. However, we were unable to clearly identify the most discriminative parameters using this approach. Therefore, we used an in-house ‘wrapper’ approach [44], which incorporates the ILP learning process in the parameter selection process, and allows the identification of more complex relationships. As an example, we can cite the disorder probability is important for the characterization of deleterious mutations, but not for neutral mutations, and is in fact the most discriminative parameter in the ILP rules. We used the wrapper approach to evaluate the quality of the parameters.

First, we estimated the discriminative power of the parameters by calculating:

$$x=\sum \left|f_{n_{i}}-f_{p_{i}}\right|$$

Where:

$$f_{n_{i}}=\mathrm{Relative}\;\mathrm{frequency}\;\mathrm{of}\;\mathrm{negative}\;\mathrm{examples}\;\mathrm{for}\;\mathrm{each}\;\mathrm{value}\;i\;\mathrm{of}\;a\;\mathrm{given}\;\mathrm{parameter}$$

$$f_{p_{i}}=\mathrm{Relative}\;\mathrm{frequency}\;\mathrm{of}\;\mathrm{positive}\;\mathrm{examples}\;\mathrm{for}\;\mathrm{each}\;\mathrm{value}\;i\;\mathrm{of}\;a\;\mathrm{given}\;\mathrm{parameter}$$

Second, we calculated the correlation matrix between all the parameters and defined a ‘correlation cutoff’, above which we considered two parameters to be significantly correlated. Third, we sorted the parameters by their discriminative power x, and for each parameter, we eliminated the significantly correlated parameters with lower x. Finally, we eliminated the parameters that had values of x lower than a given ‘x cutoff’, since these can be considered to be non-discriminating and eliminating them reduces the computational costs associated with large numbers of parameters. We tested different ‘correlation cutoffs’ in the range 0.5-0.9 with ‘x cutoff’ in the range 0.2-0.3, and measured the ILP prediction accuracy using a 10 fold cross-validation. The best performance (accuracy = 0.78) was achieved with a correlation cutoff of 0.6/0.7 and a x cutoff of 0.2 (data not shown). The correlation matrix and the reduced set of selected parameters are provided in Additional files 2 and 3.

One of the major concerns in such machine learning strategies is the problem of over-fitting. If the learned model is over-fitted to the training set, it will generally have poor predictive performance on the test data. In order to detect any potential over-fitting, we plotted the training accuracy and the testing accuracy as a function of the number of parameters in the model (Additional file 4). For a correlation cutoff <0.8, both the training and the testing accuracy increase with an increased number of parameters, indicating that the model is not over-fitted and thus, that the wrapper approach is effective. A small loss of training accuracy is observed however, for larger sets of parameters, corresponding to correlation cutoffs > =0.8.

## Results

The first part of this study was aimed at identifying a comprehensive set of parameters that may usefully characterize the structural or functional consequences of a NFS-Indel in a protein sequence. We started with the existing parameters in the SM2PH knowledge base, originally used to characterize nsSNPs. It is assumed that 83% of the harmful nsSNPs affect protein stability. For this reason, the previously developed nsSNP prediction methods have focused on information about the structure and function of the protein, as well as the conservation and the physico-chemical properties of the change implied by the mutation [31]. Using the same philosophy, we estimated or retrieved various data from SM2PH related to the sequence, structure and function of the protein and/or of the inserted/deleted amino acids. We then identified a number of additional parameters, designed specifically for the characterization of NFS-Indels, such as the probability of disordered regions in the proteins or the local perturbation of physico-chemical properties caused by the mutation.

We then used this large set of parameters to develop a machine learning strategy to study the relationships between NFS-Indels and their corresponding phenotypes. The strategy involves (i) the construction of a large data set for training and testing and the comprehensive annotation of the examples in the data set with our diverse set of structural, functional and evolutionary parameters, (ii) the optimization of an efficient Inductive Logic Programming (ILP) method to learn a set of rules that distinguish between positive (deleterious) and negative (neutral) examples and (iii) the exploitation of these rules to extract knowledge about the phenotypic effects of unknown NFS-Indels.

### Construction and annotation of the NFS-Indel data set

The background knowledge used for the machine learning strategy consists of a large data set of positive and negative examples of NFS-Indels. The construction of a suitable background knowledge base is perhaps the most important step in the development of any prediction method. Our data set consists of 757 disease-causing/deleterious NFS-Indels (108 insertions and 649 deletions) and 757 neutral NFS-Indels (101 insertions and 657 deletions), collected from publically available databases.

### Investigation of NFS-Indel pathogenicity

**Figure 1:**
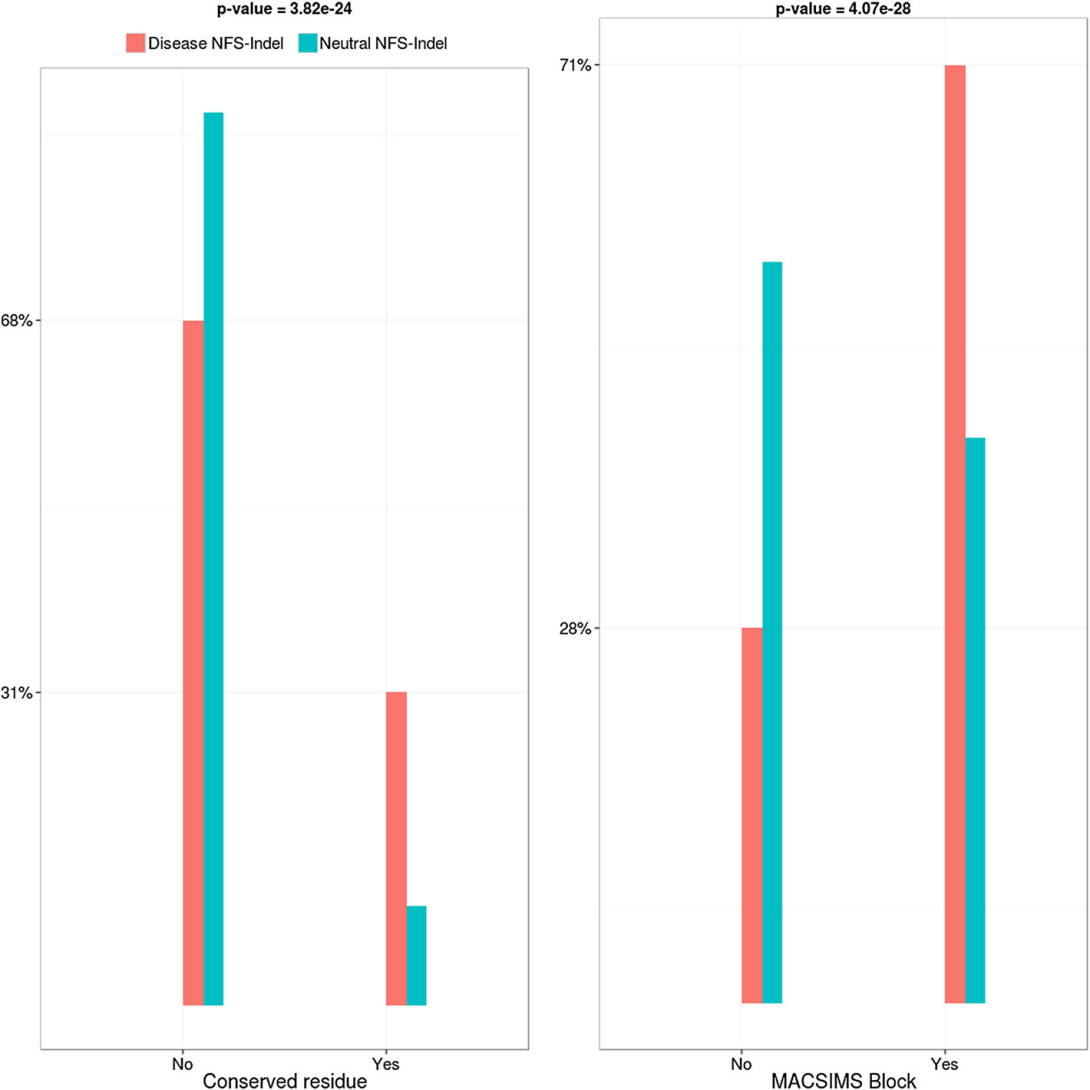
**Comparison of conservation parameters for disease-causing and neutral NFS-Indels.** P-value for chi-squared test, where the null hypothesis is that there is no significant difference the values of the parameter for deleterious and neutral variants.

**Figure 2:**
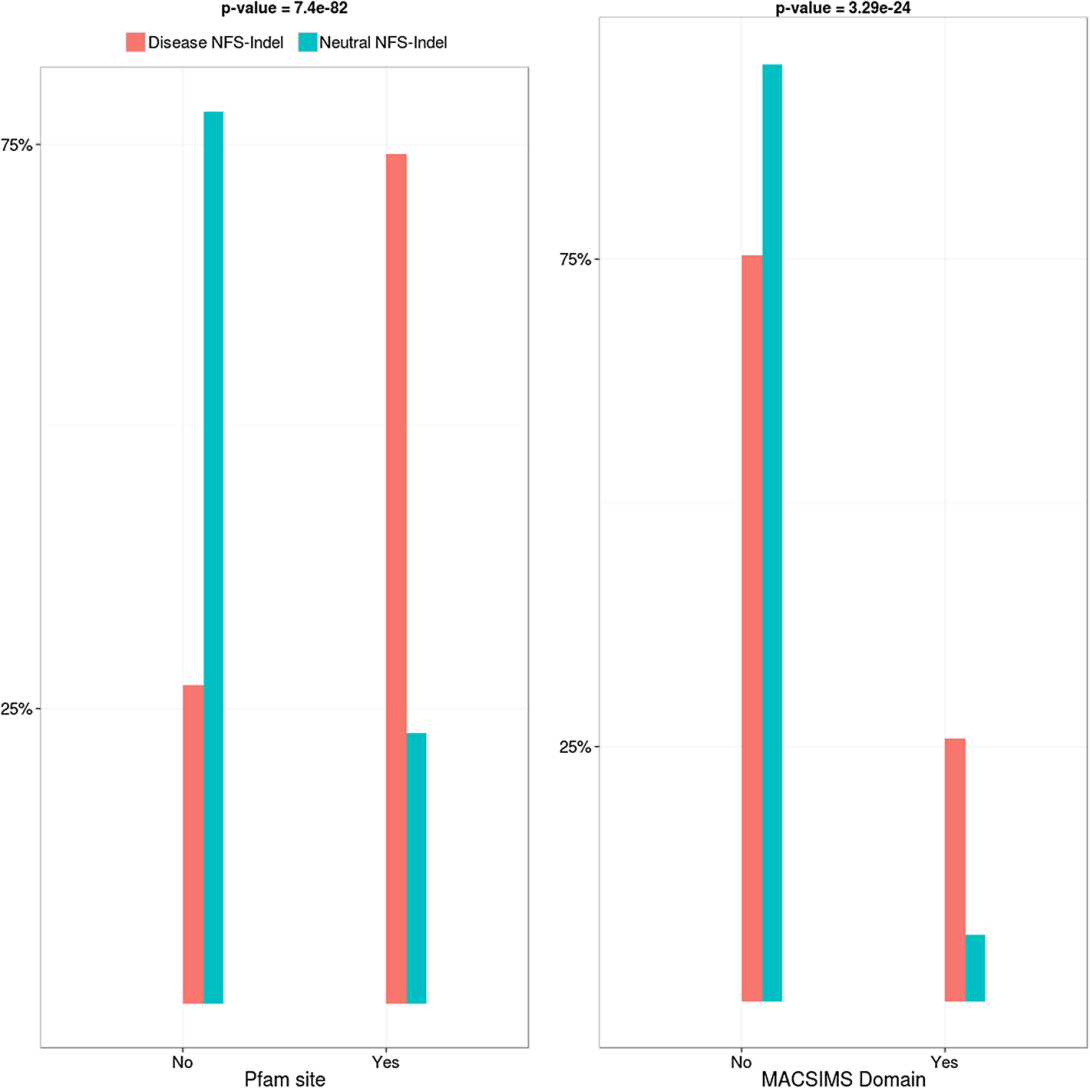
**Comparison of functional site parameters for disease-causing and neutral NFS-Indels.** P-value for chi-squared test, where the null hypothesis is that there is no significant difference the values of the parameter for deleterious and neutral variants.

**Figure 3:**
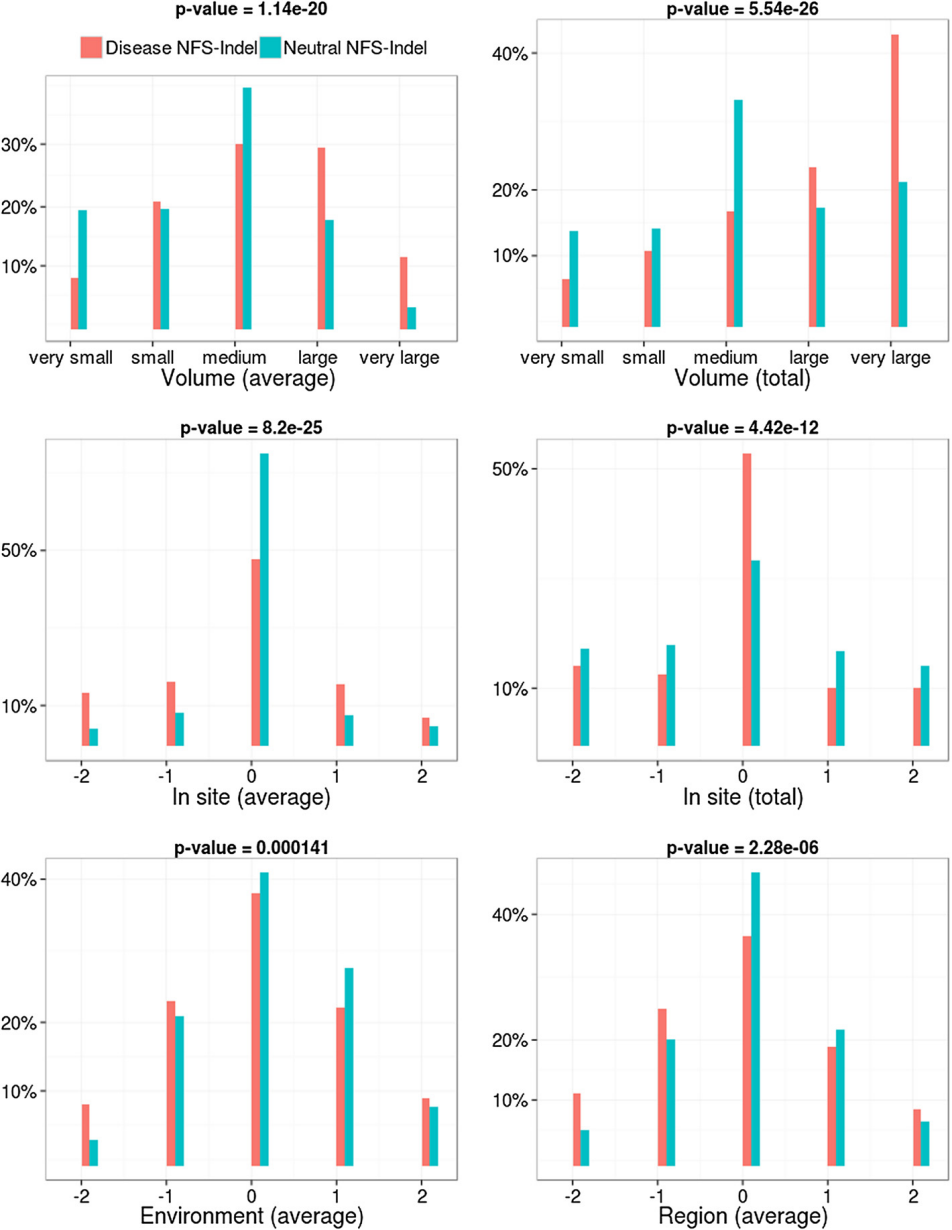
**Comparison of amino acid volumes for disease-causing and neutral NFS-Indels.** Top: volumes of the amino acids in the NFS-Indel. Middle and bottom: local perturbation of amino acid volumes caused by the NFS-Indel. P-value for chi-squared test, where the null hypothesis is that there is no significant difference between the values of the parameter for deleterious and neutral variants.

**Figure 4:**
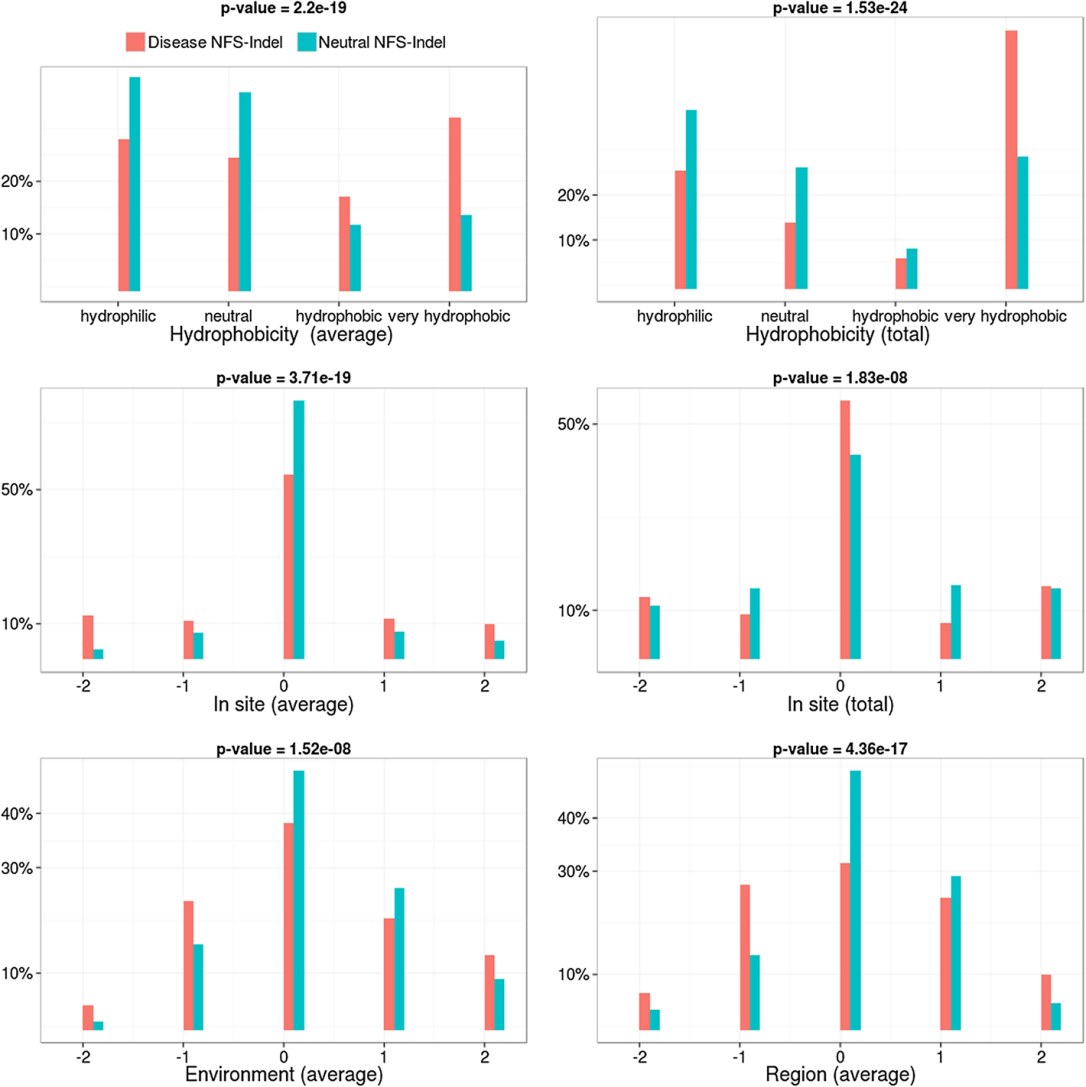
**Comparison of hydrophobicity for disease-causing and neutral NFS-Indels.** Top: hydrophobicity in the NFS-Indel. Middle and bottom: local perturbation of hydrophobicity caused by the NFS-Indel. P-value for chi-squared test, where the null hypothesis is that there is no significant difference between the values of the parameter for deleterious and neutral variants.

**Figure 5:**
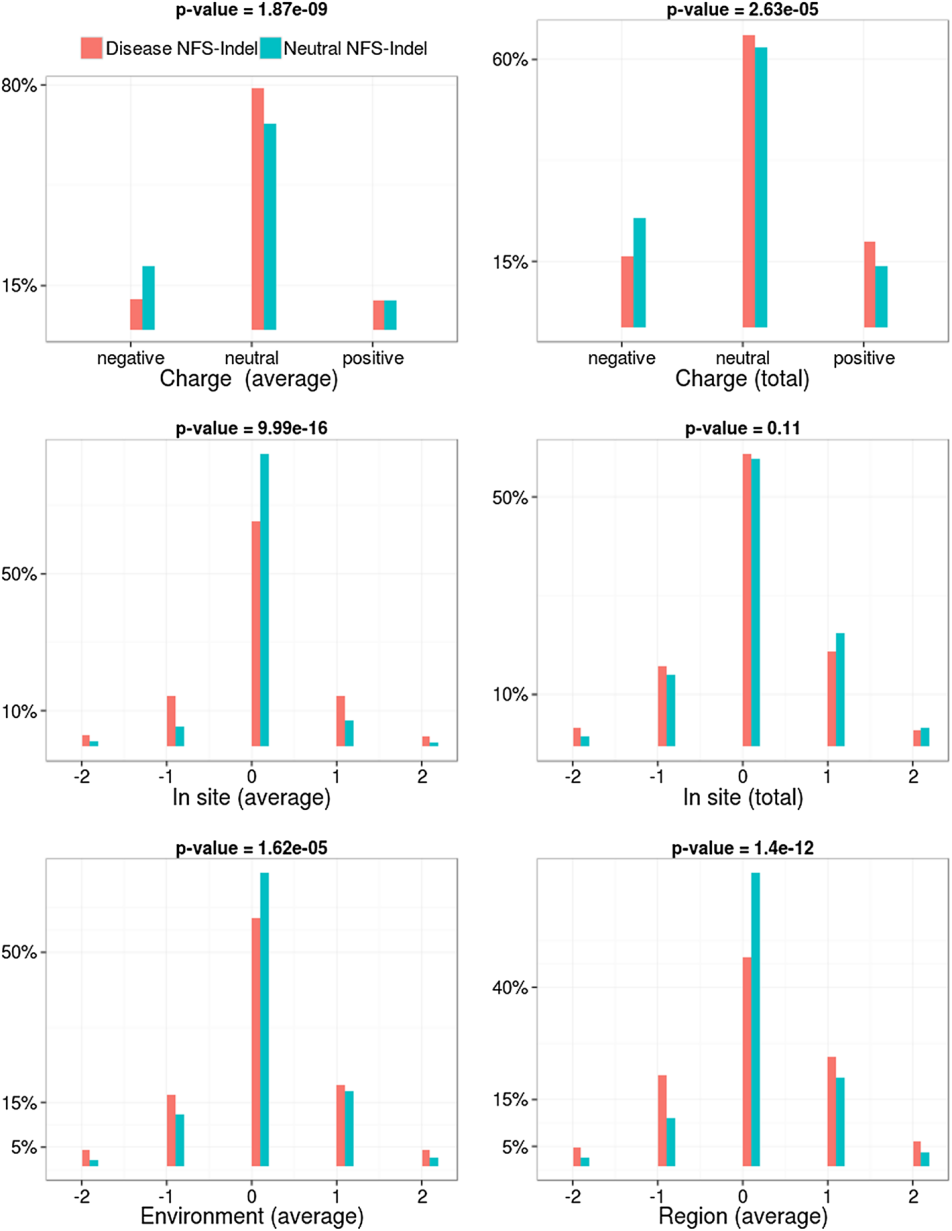
**Comparison of polarity for disease-causing and neutral NFS-Indels.** Top: polarity in the NFS-Indel. Middle and bottom: local perturbation of polarity caused by the NFS-Indel. P-value for chi-squared test, where the null hypothesis is that there is no significant difference between the values of the parameter for deleterious and neutral variants.

**Figure 6:**
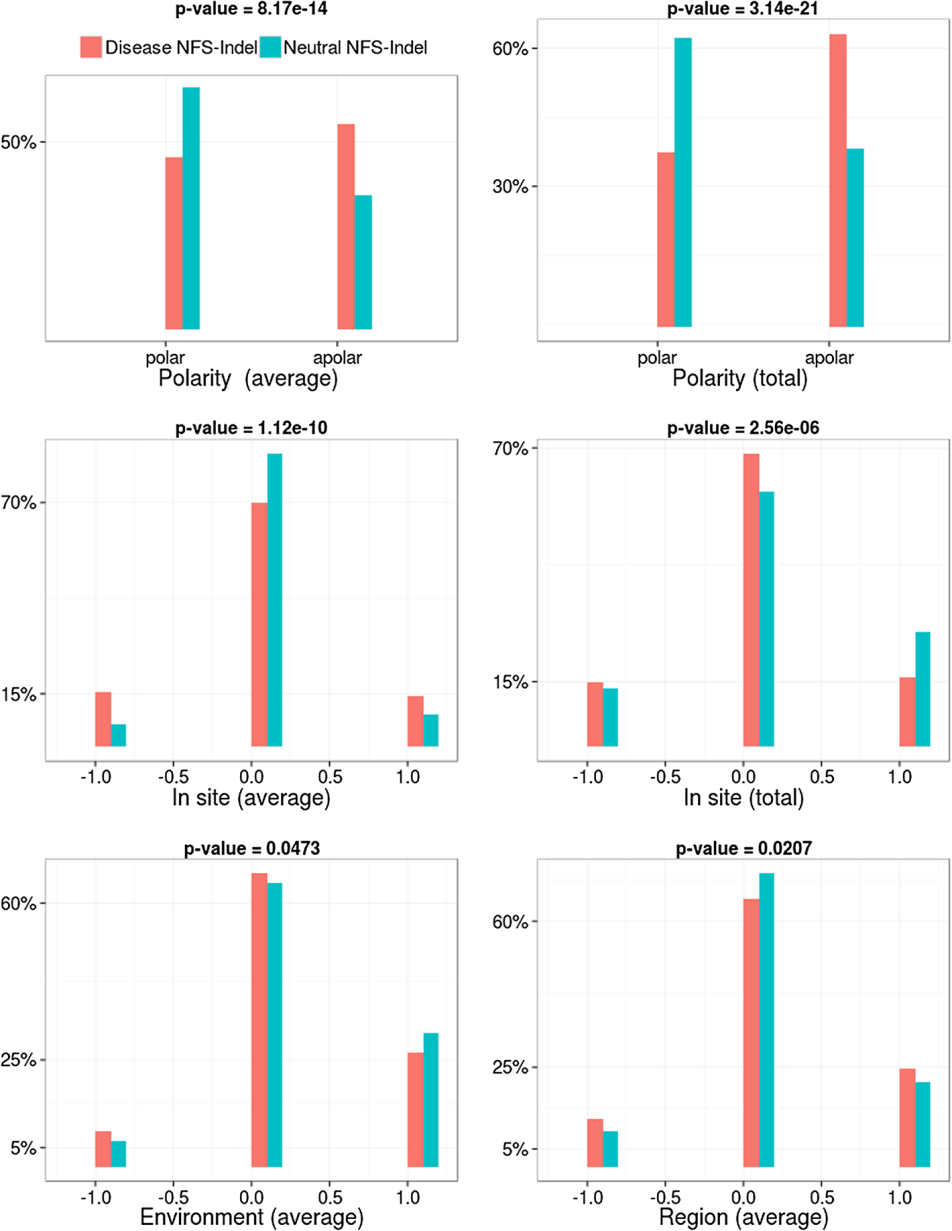
**Comparison of charge for disease-causing and neutral NFS-Indels.** Top: charge in the NFS-Indel. Middle and bottom: local perturbation of charge caused by the NFS-Indel. P-value for chi-squared test, where the null hypothesis is that there is no significant difference between the values of the parameter for deleterious and neutral variants.

**Figure 7:**
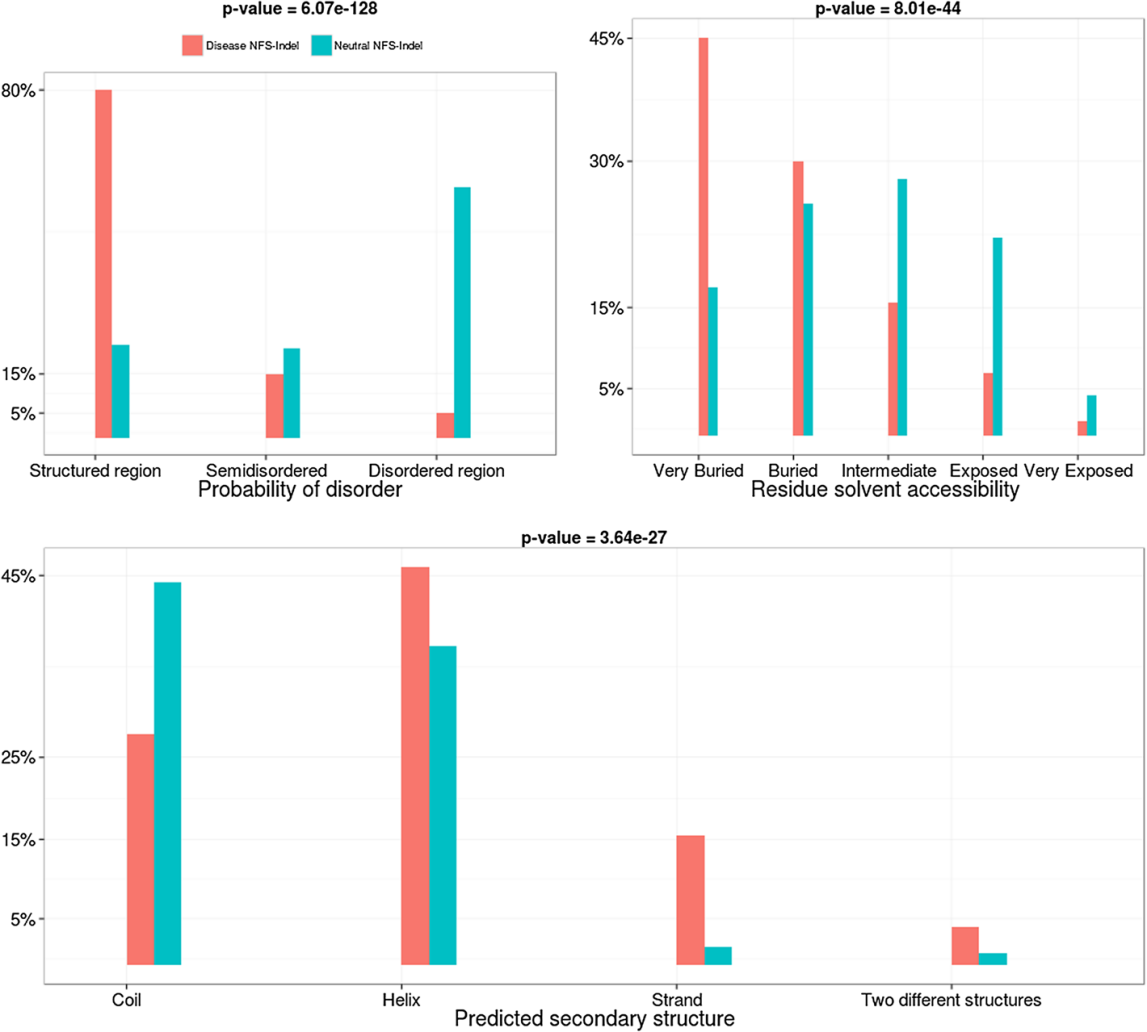
**Comparison of structural parameters for disease-causing and neutral NFS-Indels.** P-value for chi-squared test, where the null hypothesis is that there is no significant difference between the values of the parameter for deleterious and neutral variants.

**Figure 8:**
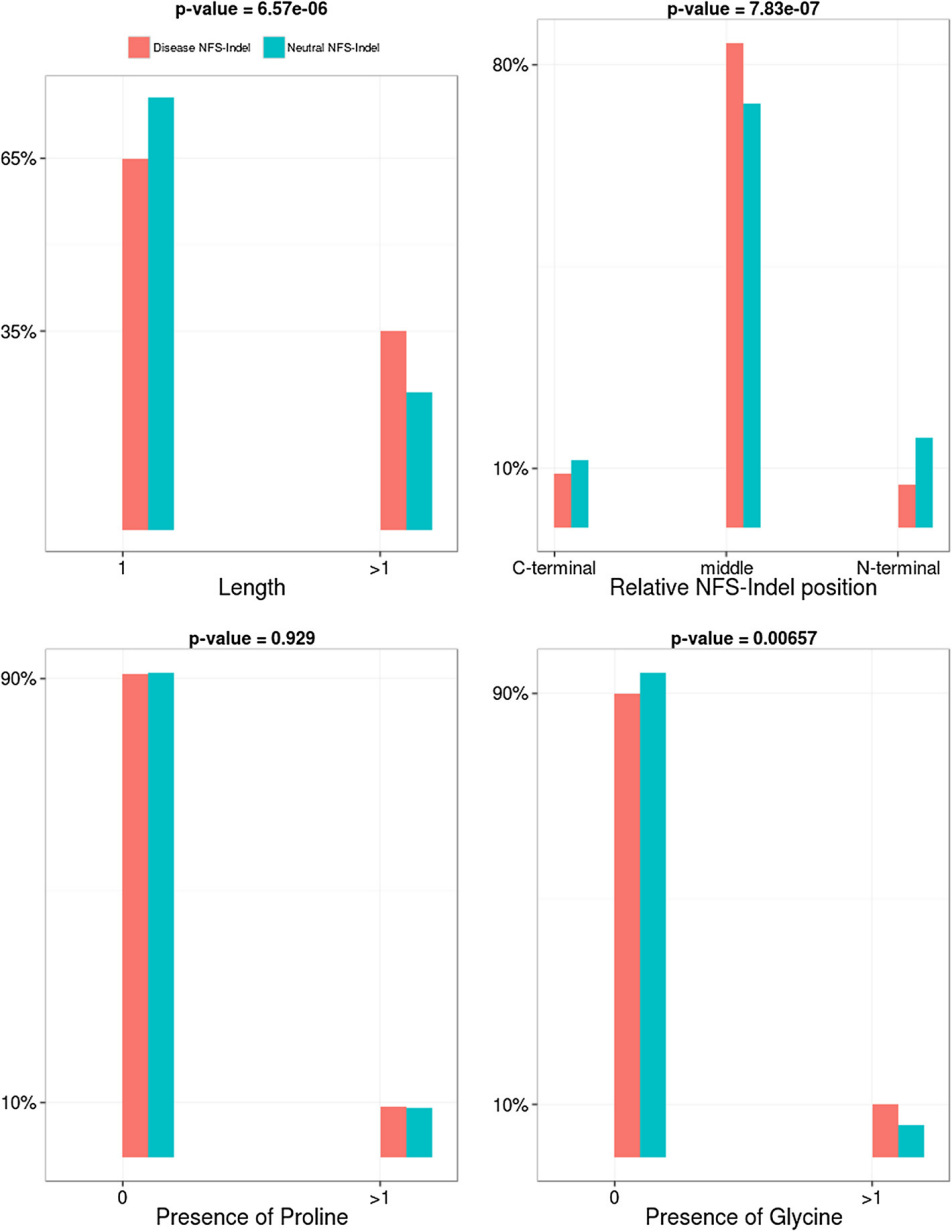
**Comparison of other parameters for disease-causing and neutral NFS-Indels.** P-value for chi-squared test, where the null hypothesis is that there is no significant difference between the values of the parameter for deleterious and neutral variants.

In this section, we aim to deduce and describe some of the general reasons behind the pathogenicity of NFS-Indels in Mendelian diseases. In order to discover differences between deleterious and neutral NFS-Indels, we performed a chi-square test (95% confidence) for each parameter, where the null hypothesis is that there is no significant difference between the values of the parameter for deleterious and neutral variants. The results of these analyses are described below:

• *Conservation:* As shown in Figure 1, disease-causing NFS-Indels are more likely to occur at conserved sites than neutral NFS-Indels. This is true for both conserved single residue positions and conserved core blocks, although the difference is more significant for the ‘core block’ parameter extracted from the MACSIMS program.

• *Functional annotations:* As shown in Figure 2, disease-causing NFS-Indels are also more likely to occur at known functional sites. The most significant difference is observed for protein domains extracted from the Pfam database.

• *Physico-chemical properties and their local perturbation:* The 4 physico-chemical parameters associated with the amino acids in the NFS-Indel, as well as their perturbation in the local environment, are shown in Figures 3, 4, 5 and 6 (amino acid volume in Figure 3, hydrophobicity in Figure 4, polarity in Figure 5 and charge in Figure 6) for both average and total scores. The results are similar for the four properties and the two types of scores (average/total):

• *Physico-chemical properties:* Disease-causing NFS-Indels tend to be bigger, more hydrophobic, and more apolar than neutral NFS-Indels. The differences are more significant for the total values compared to the average values (higher p-values). For the total values, we also observe that deleterious NFS-Indels tend to be more positively charged than neutral Indels.

• *Local Perturbation:* The perturbation parameters reflect differences between the NFS-Indel and the flanking amino acids. Here, we consider the physico-chemical parameters of the NFS-Indel compared to: the site, the environment and the region (see Methods for details). Note that a negative perturbation value indicates that the average (total) value for the amino acids in the NFS-Indel is larger than for the surrounding residues and a positive value indicates a smaller average (total) NFS-Indel parameter. In general, disease-causing NFS-Indels tend to be more associated with positive and negative changes, while neutral NFS-Indels tend to have similar physico-chemical properties compared to the surrounding site. Nevertheless, we observe some exceptions, for example, for positive changes in the volume parameter, i.e. NFS-Indels that are smaller than the surrounding amino acids, the phenotype is more likely to be neutral. It should be noted that, in the case of polarity, the differences are less significant, compared to the other physico-chemical parameters.

• *Structural annotations:* As shown in Figure 7, neutral NFS-Indels tend to be exposed on the surface of the protein, in disordered regions and in coiled coils, while disease-causing NFS-Indels are more likely to be located in structured, buried regions and secondary structures. Concerning the type of secondary structure, deleterious NFS-Indels are more often found in strands than in helices.

• *Others:* As shown in Figure 8, deleterious NFS-Indels are more likely to be located in the central region of the protein and are longer than neutral NFS-Indels. No significant differences are observed when the NFS-Indel includes a proline, but deleterious NFS-Indels tend to contain a glycine more often than neutral NFS-Indels.

### Machine learning method and prediction performance

We used a 10-fold cross validation experiment to estimate the accuracy of our method, where the complete data set was randomly divided into 10 parts (nine parts for training, the rest for testing) and the process was repeated 10 times. For the 10 tests, our computational strategy achieves an average prediction accuracy on the test set of 79%, sensitivity of 89%, specificity of 69%, precision of 75%, negative prediction value of 85%, and Matthews Correlation Coefficient (MCC) of 0.59 (Table 5). We then identified the set of rules produced by the ILP algorithm that achieved the highest accuracy (83% on the test set, corresponding to fold 1). This final rule set is composed of 241 human-interpretable rules that represent new knowledge about the molecular perturbations underlying disease-causing NFS-Indels. The complete list of parameters used in this set are indicated in Table 1.

**Table 5:** Validation of KD4i by 10 fold cross-validation

**Fold**	**Accuracy**	**Sensitivity**	**Specificity**	**Precision**	**NPV**	**MCC**
0	77%	87%	67%	76%	81%	0.55
1	83%	93%	74%	78%	91%	0.68
2	78%	85%	71%	76%	81%	0.56
3	79%	96%	63%	72%	94%	0.62
4	78%	88%	68%	74%	85%	0.57
5	73%	86%	60%	67%	82%	0.48
6	77%	85%	71%	73%	83%	0.56
7	82%	90%	74%	79%	87%	0.65
8	83%	85%	81%	83%	83%	0.66
9	79%	90%	65%	76%	85%	0.58
Average	79%	89%	69%	75%	85%	0.59

NPV: negative prediction value, MCC: Matthews Correlation Coefficient.

Finally, we compared the performance of KD4i with two recent methods for the prediction of the phenotypic effects of NFS-Indels, namely DDIG-in and SIFT-Indel. DDIG-in uses a Support Vector Machine (SVM) as the learning method, while SIFT-Indel uses a rule set derived from a decision tree algorithm. In order to provide a more direct comparison with the DDIG-in method, we have also trained a SVM on our dataset and parameters. The results are shown in Table 6. We observe that the accuracies obtained with DDIG-in and our SVM implementation are similar, despite the fact that the size of the data set used to train the DDIG-in algorithm is almost 4 times larger than the one used here, since we used only publically available data in our experiment. In comparison to SIFT-Indel, the KD4i ILP approach (based on the final rule set) achieves similar accuracy, but with higher sensitivity and lower specificity.

**Table 6:** Comparison of prediction performance for KD4i with the DDIG-in and SIFT-Indel methods

**Algorithm**	**Accuracy**	**Sensitivity**	**Specificity**	**Precision**	**NPV**	**MCC**
DDIG-in	83	-	-	-	-	0.67
SIFT-Indel	82	81	82	82	-	0.63
KD4i (SVM)	84	80	87	88	79	0.67
KD4i (ILP) (average)	79	89	69	75	85	0.59
KD4i (ILP) (final)	83	93	74	78	91	0.68

For the KD4i (ILP) implementation, the average performances in the 10-fold cross-validation and the performance of the final selected rule set (corresponding to fold 1 in Table 5) are provided. For DDIG-in and SIFT-Indel, the performances for combined insertions and deletions (as originally reported by the authors, for similar balanced data sets) are shown.

The higher sensitivity of KD4i is due in part to the power of the ILP approach, but also to the parameters used in the final set of rules. Indeed, all the tools tested here use different sets of parameters to characterize the NFS-Indels. As shown on Table 7, the disorder probability and the sequence conservation are important parameters for all three methods. However, the KD4i rule set includes other parameters that are used in only one or the other of the existing methods, such as the solvent accessible surface area (also used in DDIG-in) or location in a Pfam functional domain (also used in SIFT-Indel). In addition, we have highlighted the importance of some novel parameters, notably the local perturbation induced by the variant, in terms of amino acid volume for example.

**Table 7:** Top parameters used in KD4i (ranked according to their percent usage in the rules), DDIG-in and SIFT-Indel

**KD4i**	**DDIG-in**	**SIFT-Indel**
Disorder Probability	Probability of disorder	Fraction of Pfam domains
Indel in a Pfam domain	Solvent accessible surface area	Indel in a repeat
Conserved amino acid	DNA conservation score 1	Indel in a disordered region
Relative solvent accessible surface area	DNA conservation score 2	DNA conservation score
Local perturbation in volume (average)	Sheet/amino acid conservation score 1	-

### Assessing the rules produced by KD4i

KD4i is a rule-based system and the output is a prediction in binary form (deleterious/neutral). One major advantage of our approach is that the rules used to predict deleterious NFS-Indels are available in a human-interpretable format. In addition to providing useful information about the pathogenicity of specific NFS-Indels, it is possible to consider the prediction performance of a given rule independently of the rest. In order to identify the best performing rules (i.e. those resulting in predictions with the highest confidence), we ranked them according to two characteristics: coverage and precision.

Coverage is defined as the percentage of deleterious NFS-Indels in the training set that can be explained by a given rule. Given that we have 757 deleterious (positive) NFS-Indels in our data set and we have defined 241 rules, the expected average coverage is 3.2%. Values below this cutoff thus reflect rules that are more specific for a particular type of NFS-Indel, while larger values reflect more general rules. The average coverage of our rules is 2.8%, reflecting a slight tendency towards more specific rules. The maximum coverage is 7.4% and the minimum 0.8%.

**Figure 9:**
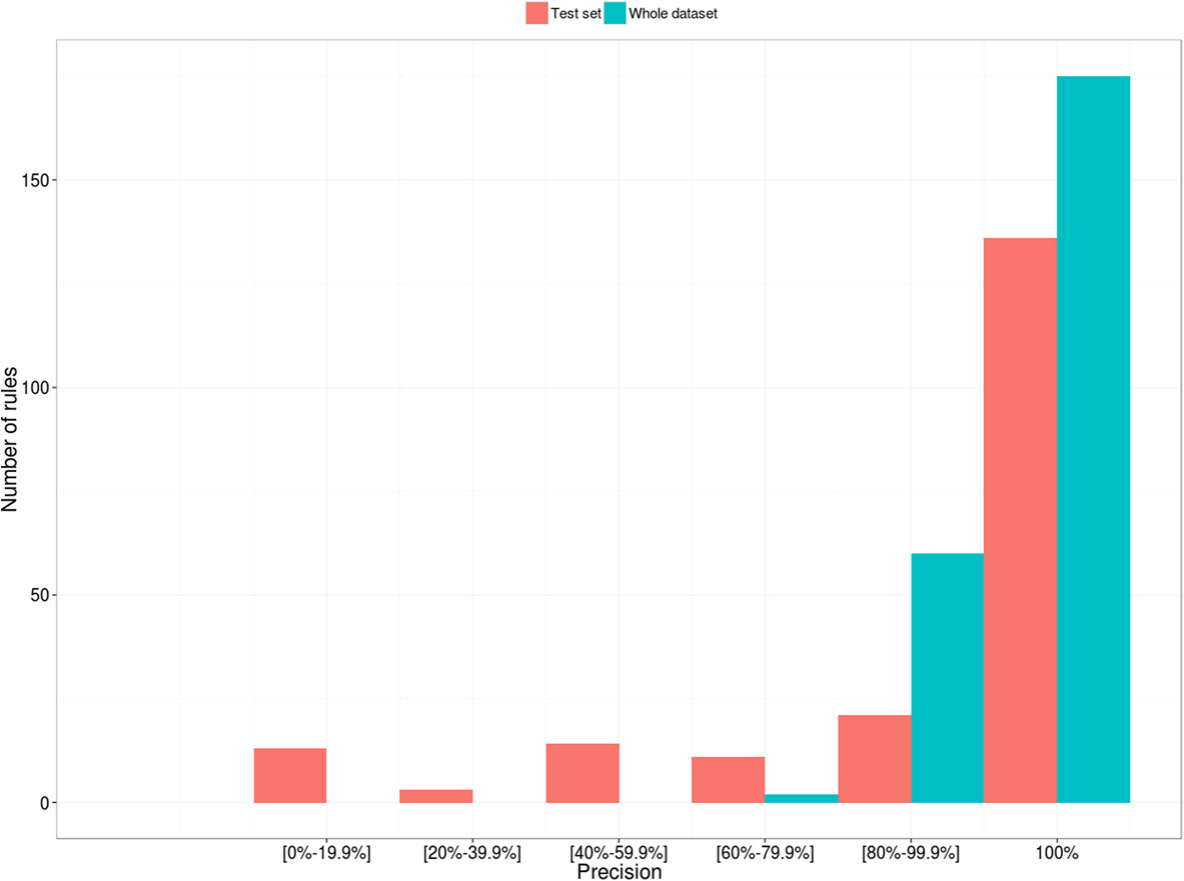
Precision of rules in the final selected rule set calculated for the whole data set and for the test set.

Precision (also known as positive predictive value) is the proportion of positive test results that are true positives and reflects the probability that a mutation predicted to be deleterious is truly deleterious. For two rules with the same precision, we consider the best rule to be the one that has higher coverage. Since we set the noise option in the ILP algorithm to 0 (i.e. the rules cannot cover any negative examples), the precision of the rules in the training set is 100%. Figure 9 shows the precision of the rules calculated for the complete data set and for the test set only. If the test set is representative of the whole data set, the precision values should be similar. As expected, the precision of the rules observed in the test set is generally slightly lower than for the whole set, with less rules achieving 100% precision. The precision should be increased when the size of whole data set is increased and the precision in the test set should tend towards that observed for the whole data set, as was demonstrated in [45].

The complete list of ranked rules is provided in Additional file 5, together with their coverage and precision statistics, and the top ranking rules are discussed in the following section.

Another way to estimate the most reliable predictions is to identify the NFS-Indels that are predicted to be deleterious by more than one rule. Table 8 shows the average precision as a function of the number of rules that cover a given prediction. We observe that the precision generally increases as the number of rules increases.

**Table 8:** Precision of predictions as a function of the number of associated rules

**a) → Whole data set**			
**N° of rules**	**N° of deleterious NFS-Indels covered**	**N° of neutral NFS-Indels covered**	**Average precision**
1	27	5	84.38
2	26	7	78.79
3	25	1	96.15
4	16	1	94.12
5	14	3	82.35
6	13	1	92.86
7	220	2	99.01
**b) → Test set**			
**N° of rules**	**N° of deleterious NFS-Indels covered**	**N° of neutral NFS-Indels covered**	**Average precision**
1	8	5	61.54
2	6	7	46.15
3	6	1	85.71
4	8	1	88.89
5	4	3	57.14
6	3	1	75.00

### Rules governing NFS-Indel pathogenicity

The KD4i method provides explicit rules that shed light on the reasons for the pathogenicity of specific NFS-Indels. In this section, we describe the three top ranking rules in the final rule set identified above (corresponding to fold 1).

1. Rule 20 (coverage 7.26%, precision 100%):

deleterious (A) if secondary_ structure (A, strand), block (A, true), local_perturbation_region_polarity_total (A, equal), relative_indel_position (A, middle).

This rule can be interpreted as: a NFS-Indel (A) is deleterious if it is located in a beta strand and in a conserved block, the polarity of the NFS-Indel is equal to that of the local region and the NFS-Indel is found in the central region of the protein. The rule thus indicates the specific conditions that determine NFS-Indel pathogenicity in beta strands. Indeed, we have observed that deleterious NFS-Indels occur more often than neutral NFS-Indels in secondary structure elements (Figure 7) and in particular in beta strands, probably due to their highly organized structure. However, KD4i is able to explain in more detail some of the reasons underlying this difference. In fact, almost half of the deleterious NFS-Indels in strands are found in the central region of the protein and in a conserved block. The prediction is completed by the specification of the local perturbation of the polarity in the region.

2. Rule 14 (coverage 7.00%, precision 100%):

deleterious (A) if pfam (A, true), domain (A, true), conserved_residue (A, false), local_perturbation_region_polarity_total (A, equal), indel_hydrophobicity_total (A, very_hydrophobic).

This rule describes NFS-Indels that are located within a Pfam or Uniprot domain, but do not affect a conserved residue. In this case, a very hydrophobic NFS-Indel can still have a deleterious effect on the protein structure or function.

3. Rule 19 (coverage 6.80%, precision 100%):

deleterious (A) if block (A, true), probability_of_disorder (A, structured), local_perturbation_region_polarity_total (A, equal), indel_hydrophobicity_total (A, very_hydrophobic), local_perturbation_in_site_volume_average (A, two_more).

The rule describes a NFS-Indel in a conserved block that is situated in a structured region of the protein. Here, the insertion/deletion of very hydrophobic and very large residues probably disrupts the organization of the local region.

The complete set of rules is provided in the Additional file 5.

### Assessment of the reliability of the KD4i predictions

**Figure 10:**
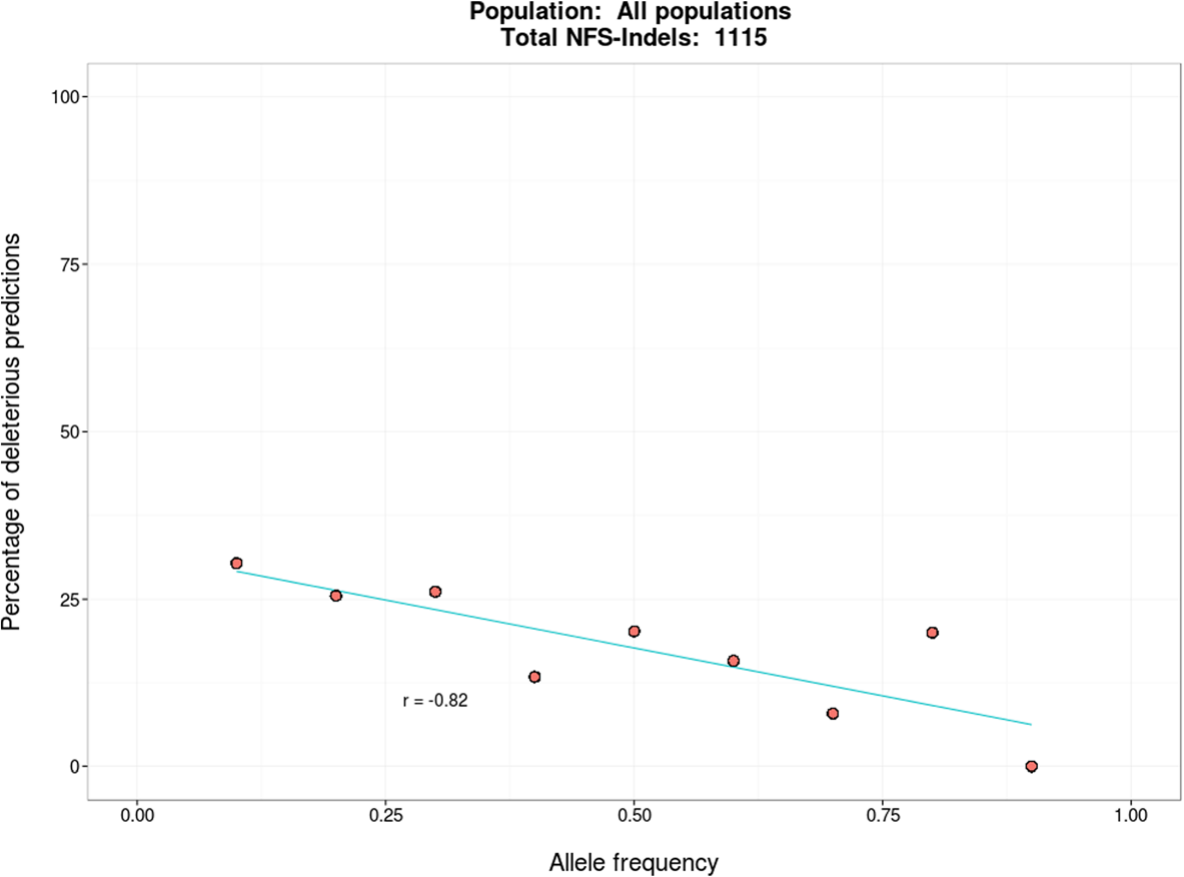
Correlation of the percentage of KD4i deleterious predictions with the allele frequencies of NFS-indels found in the 1000 Genome Project.

In order to assess the reliability of our predictions, we analyzed population data from the 1000 Genomes Project [46], including genomes of 1,092 healthy individuals from 14 populations. The majority of the variants observed in the 1000 Genomes are therefore expected to be neutral. However, several studies [47,48] have shown that healthy individuals may carry deleterious variants without any obvious phenotypic effects. As an example, Watson’s genome [49] has a well-known Alzheimer’s variant without apparent clinical effect. Nevertheless, these variants are expected to be observed with low frequencies at the population level. Conversely, variants found with higher frequencies are less likely to be deleterious. Therefore, we grouped the NFS-Indels in the 1000 Genomes by allele frequency (at 0.1 intervals) and compared these frequencies with the predictions produced by KD4i (Figure 10). As might be expected, the percentage of variants predicted to be deleterious decreases as the allele frequency increases (r = −0.82), thus confirming the pertinence of our predictions.

### Case study: NFS-Indel in a kinesin protein

To illustrate the potential of the KD4i approach to infer new knowledge about individual variants, we have chosen a variant from the test set (fold 1), namely a deletion of an asparagine residue at position 526 (N526del) of the KIF5A protein. KIF5A (UniProt ID: Q12840) belongs to the microtubule-associated protein family of kinesins, that serve as molecular motors to distribute intracellular cargo along microtubules. KIF5A is expressed exclusively in neurons, and has been recently linked to hereditary spastic paraplegias (HSPs), a genetically heterogeneous group of neurodegenerative disorders characterized by progressive lower-limb spasticity and weakness. The HSP pathology is characterized by axonal degeneration of motor and sensory neurons [50].

The variant (**KIF5A_**N526del) is predicted to be deleterious by 7 rules. We can compute the total precision, by taking the true/false positives for each rule and applying:

$$\mathrm{Precision}\;\mathrm{total}=\sum \frac{\mathrm{True}\;\mathrm{positives}}{\mathrm{True}\;\mathrm{positives}+\mathrm{False}\;\mathrm{positives}}$$

Since the precision is 100% and we have a large number of rules, we have high confidence in the prediction.

**Figure 11:**
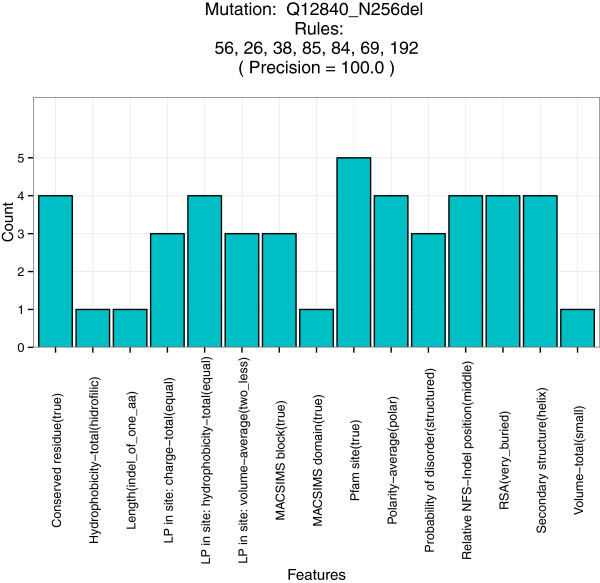
Histogram of occurrence of the different parameters in the rules that cover the kinesin heavy chain isoform 5A (Uniprot ID: Q12840) N256 deletion.

In order to extract more detailed information, we constructed a histogram of the usage of each parameter in this set of 7 rules. As shown in Figure 11, conservation-related (conserved residue and MACSIMS domain and block) and functional (Pfam site) parameters are largely represented. In addition, since the amino acid deleted is polar, hydrophilic and small, we hypothesize that the variant does not disrupt the structural folding process. We can conclude at this point that the deletion is probably a loss-of-function variant.

In the literature, the mutated protein, KIF5A**_**N526del is described as stable, but nonfunctional [50]. The mutation was subsequently shown to decouple microtubule binding of the motor, leading to inactivation of ATPase. Interestingly, drosophila larvae harboring either homozygous or hemizygous mutations in homologous genes of KIF5A exhibit aberrant neuronal intracellular anterograde trafficking of membranous organelles and accumulation in so-called “organelle jams”, that leads to neurodegeneration [51].

## Discussion and conclusions

Here, we have described a computational system, KD4i, which exploits a machine learning strategy, namely ILP, to extract information and generate new knowledge about NFS-Indel pathogenicity. A major advantage of our approach is the ability to construct a model that provides putative explanations for the NFS-Indel pathogenicity, thanks to the production of human-interpretable rules. The model constructed by our method includes 207 rules that explain NFS-Indel pathogenicity in terms of problems related to stabilization of structured regions or loss of functional sites, for example. In addition, we have identified new parameters that are useful in discriminating between disease-causing and neutral NFS-Indels. The probability of disorder is the most discriminating parameter for NFS-Indel pathogenicity, since NFS-Indels in structured regions are very frequently disease-causing. This can be combined with other parameters, such as the location of the NFS-Indel in a conserved segment (core block) of the protein. Other predictors of disease-causing NFS-Indels include the perturbation of local physico-chemical properties, such as hydrophobicity or amino acid volume.

In the future, in order to improve the accuracy KD4i, we will address the optimization of the parameters, extend the data set to include more representative NFS-Indels and investigate new parameters that can be used to better characterize the variants. Furthermore, we intend to combine the results of the ILP (deleterious/neutral) with other machine learning methods (such as SVM or decision trees) into a single ‘consensus’ prediction. This will hopefully improve the prediction accuracy. Finally, we hope to expand our infrastructure to cover other types of variants, for example those occurring outside the protein coding regions (promoter, 5-UTR, 3-UTR, etc.) or in non-coding regions (RNA genes, regulatory sites, etc.), given that these regions account for ~88% of trait-associated variants [52].

## Competing interests

The authors declare that they have no competing interest.

## Authors’ contributions

CBDN carried out the parameter analysis, designed the machine learning algorithm and drafted the manuscript. HN participated in the statistical analysis and design of the machine learning algorithm. OP participated in the design of the study and the interpretation of the results. JDT conceived the study, participated in its design and helped draft the manuscript. All authors read and approved the final manuscript.

## Authors’ information

Carlos Bermejo-Das-Neves and Hoan-Ngoc Nguyen are joint first authors.

## Supplementary Material

Additional file 1 Complete set of variants used in the final data set. Click here for file

Additional file 2 **Correlation matrix.** Microsoft Excel file. Click here for file

Additional file 3 **Reduced set of uncorrelated parameters.** Microsoft Excel file. Click here for file

Additional file 4 **Training and testing accuracies versus number of parameters.** Microsoft Excel file. Click here for file

Additional file 5 **Rule set ranked by precision and coverage.** Microsoft Excel file. Click here for file
